# Modern Aspects of the Treatment of Graves' Disease

**Published:** 1894-03-17

**Authors:** 


### Modern Aspects of the Treatment of Graves' Disease.

Everyone who has seen much of this disease allows that the treatment of it is most unsatisfactory. There is no definite information as to what is the best drug to give, such, for instance, as we have in psoriasis, for which everyone acknowledges arsenic to be by far the best remedy.

Many drugs have been tried for exophthalmic goitre. Naturally, considering the anæmia, perchloride of iron is one of them, but that was long ago discarded, for, as Wilks pointed out, it often actually does more harm than good. Perhaps out of the very long list of medicines those which have found most favour are belladonna and digitalis, the latter having been used on the recommendation of Trousseau. The very unsatisfactory results obtained by drugs have made physicians look on all sides for other modes of treatment, and some have therefore applied Leiter's coils, with ice-cold water circulating through them, to the heart, the thyroid gland, and the protruding eyes, and in certain cases with undoubted benefit; but this good result did not take place in a sufficient number of instances for the treatment to be widely adopted, nor is that surprising when we remember that some patients suffering from exophthalmic goitre recover even if left to themselves, and, on the other hand, some of those treated with Leiter's coils relapsed as soon as these were left off. For the last few years many cases have been treated by electricity. This treatment originally came into vogue because it was believed that the cervical sympathetic was diseased in exophthalmic goitre, but although the reasons which led to the employment of electricity were erroneous, it sometimes benefited the patient. The galvanic current should be used, and it should be allowed to flow for some minutes every day through the enlarged thyroid, one pole being placed on each lobe of the organ. After that has been done, a mild current being used, one pole should be placed over each eye for two or three minutes. There is no doubt that in this way slight cases may sometimes be improved.

Still more recently surgeons have been slowly and cautiously excising part of the thyroid gland in cases of exophthalmia goitre. All treatment previous to this was either quite arbitary and irrational, or was founded on an incorrect notion of the pathology of the disease. About this the wildest notions prevailed. The exophthalmia was thought to be due to a great increase of fat in the orbit; it was considered that the orbitalis muscle of Müller, which is in man very little developed, was disordered owing to some lesion of the sympathetic. In fact, the whole disease was set down to disturbance of the cervical sympathetic, for it was considered that the cervical ganglia were diseased. The cause of this error was, that sufficient attention was not paid to the wide variations these organs may attain in health in the human adult.

Gradually observers came to see that probably the disease was set up by some disturbance of the central nervous system, and the fact that Savage and others urged that it was frequently associated with mental disorder gave an impetus to this view. Next actual microscopic changes in the central nervous system, especially about the floor of the fourth ventricle, were described, and this observation was corroborated by several physicians. Still there was no clue as to treatment. It appeared clear that the symptoms of exophthalmic goitre were due to some functional disturbance of the central nervous system, accompanied in some rare cases by actual microscopic changes, but we were quite in the dark as to why this functional disturbance should occur. Then there came those brilliant researches carried on by Horsley and others, which proved that myxœdema was caused by the loss to the body of some secretion which the thyroid normally pours into the blood, and that this disease, cretinism, and probably acromegaly can be cured by the administration to patients of the thyroid of the sheep. These discoveries have given an immense impetus to the excision of the thyroid gland in exophthalmic goitre, for they render it highly probable that the functional disturbance of the central nervous system which causes the symptoms of the disease is induced by the enlarged and hypertrophied thyroid gland pouring constantly into the circulation too much of its secretion with which the patient is in fact poisoned.

Putnam has done immense service by collecting together in the *Journal of Nervous and Mental Disease* for December, 1893, all the published cases of the removal of all or part of the thyroid gland in Graves' disease. The first thing to notice is that great care must be taken in the administration of the anæsthetic, for there seems no doubt about the fact that there have been an unusual number of deaths at the operation caused by the anæsthetic, probably owing to cardiac failure, partly from the previous condition of the heart, and partly because of the many important nerves which may be wounded in the operation. Not only is there this danger, but for some few days after the operation the pulse is often rapid and weak; there is great prostration, with a weak voice, and the temperature may rise. Usually after a few days these unfavourable symptoms pass off, even if one of them, as is often the case, has been laryngeal paralysis.

In many of the cases after the first few days, the improvement began, the palpitation was less, and the eyes were not nearly so prominent; in others the improvement came on a little later. An interesting and important point is the liability of the part of the thyroid that is left behind to grow large again. In one or two instances this has happened, but its frequency is much over-estimated. The almost invariable rule is shrinkage of the remaining portion.

The character of the enlargement of the thyroid gland, whether, for instance, chiefly vascular or chiefly parenchymatous, appears to make no difference, improvement always, according to Putnam, follows the operation. It should be mentioned that in most of the cases that have been operated upon the goitre has been large and the symptoms urgent. We do not yet know whether benefit would follow in the slighter cases. Another point on which we want more information is how much of the gland should be removed. As now we know that when too much of it has been taken away the bad symptoms can probably be controlled by the administration of sheep's thyroid, many surgeons will err on the side of removing too much. Putnam has shewn that the removal of part of the thyroid is quite sufficient.

